# Giant Frontoethmoidal Encephalocele in an Infant: A Case Report

**DOI:** 10.7759/cureus.78820

**Published:** 2025-02-10

**Authors:** Elias Gallardo-Navarro, Angel Puente Sanchez, Luis Felipe Gordillo Dominguez, Ranulfo Enrique Pazos Alvarado

**Affiliations:** 1 General Surgery, Hospital Español, Mexico City, MEX; 2 Plastic and Reconstructive Surgery, Hospital Infantil de México, Federico Gómez, Mexico City, MEX; 3 Pediatric Neurosurgery, Hospital Infantil de México, Federico Gómez, Mexico City, MEX; 4 Pediatric Anesthesiology, Hospital Infantil de México, Federico Gómez, Mexico City, MEX

**Keywords:** congenital abnormalities, encephalocele with myelomeningocele, facial reconstruction, giant tumor, newborn infant

## Abstract

Giant encephalocele is a rare pediatric surgical entity that poses unique challenges. Few cases have been reported in the medical literature, and its cause is unknown. Factors that increase the likelihood of developing this pathology are radiation, infections, hyperinsulinemia, vitamin deficiencies related to neural tube closure defects, maternal smoking, alcohol, and anticonvulsants. A three-month-old female infant, with no factors associated with the current condition, with poor prenatal control, diagnosed with giant frontoethmoidal encephalocele, and with the presence of a frontal tumor of approximately 15 cm in its major axis, underwent surgery by a neurosurgeon and a plastic surgeon to correct the craniofacial deformity. The first approach was performed by removing the tumor with the closure of the meninges and skull. Then in the second surgical time, craniofacial reconstruction was performed by the plastic surgery team, using the same skin without the need for flaps of any other structure and without the presence of complications. The patient had no post-surgical complications. After seven years of medical follow-up, she has a normal staturoponderal and psychomotor development, no intellectual deficit, and adequate aesthetic results.

## Introduction

Giant encephalocele is an uncommon pathology. Currently, prenatal ultrasound studies come to detect these anomalies; however, it is reported that the incidence of encephaloceles is 1-4 cases per 10000 live births, with the occipital region being the most common site, followed by the frontoethmoidal, parietal, and sphenoid regions, respectively [1,2]. It is a congenital malformation that belongs to the group of alterations of neural tube closure characterized by a protrusion of intracranial contents due to a defect of the skull and dura mater. When the protrusion contains only meninges and cerebrospinal fluid, it is more correct to call it meningocele; however, it is common to call all cranial defects encephalocele regardless of the presence or absence of brain tissue in the sac [1,3]. Within the pathophysiology, encephaloceles result in the congenital opening of regions of the midline, just at the junction of the chondrocranium and desmocranium, that is, the base and cranial vault, respectively. This allows the meninges, the brain, or both to migrate through the established defect, which occurs due to an abnormal closure of the neural tube. This occurs between three and four weeks of gestation [3,4]. 

Previously, surgical management was performed in two phases, starting with surgery and cranial repair, followed by the external resection of the mass. As such, the surgical objective in patients with giant encephalocele is to repair the dural defect and correct the associated hypertelorism [5,6]. To correct these craniofacial complications, one must have an understanding of the facial subunits, which were described by Gonzalez-Ulloa et al. in 1954 and later by Millard in 1966, who referred to the nasal subunits, and in 1987, they were defined in more detail, to extend these concepts to reconstructive surgery of the rest of the face. Facial subunits share similar characteristics in terms of color, texture, thickness, elasticity, mobility, pore size, presence of hairs, and vasomotor response, and the face can be divided into eight units including the forehead, eyelids, nose, cheeks, ears, lip, mentonian region, and neck [6-8]. The forehead consists of three subunits, namely, central, lateral, and brow, while the nasal unit has nine subunits: the dorsum; right and left lateral walls; tip, left, and right alar walls; right and left alar base; and columella of the nose [7-9]. 

Currently, repair in a single surgical time brings benefits by not exposing the patient to two surgeries that involve extensive procedures, prolonged anesthesia, and great blood loss, being a multidisciplinary surgical option that previously was only performed to correct complications of cerebrospinal fluid leak immediately and with an unfavorable outcome for the patient. This case demonstrates the favorable aesthetic results seven years after surgery for a rare pediatric pathology. 

## Case presentation

**Figure 1:**
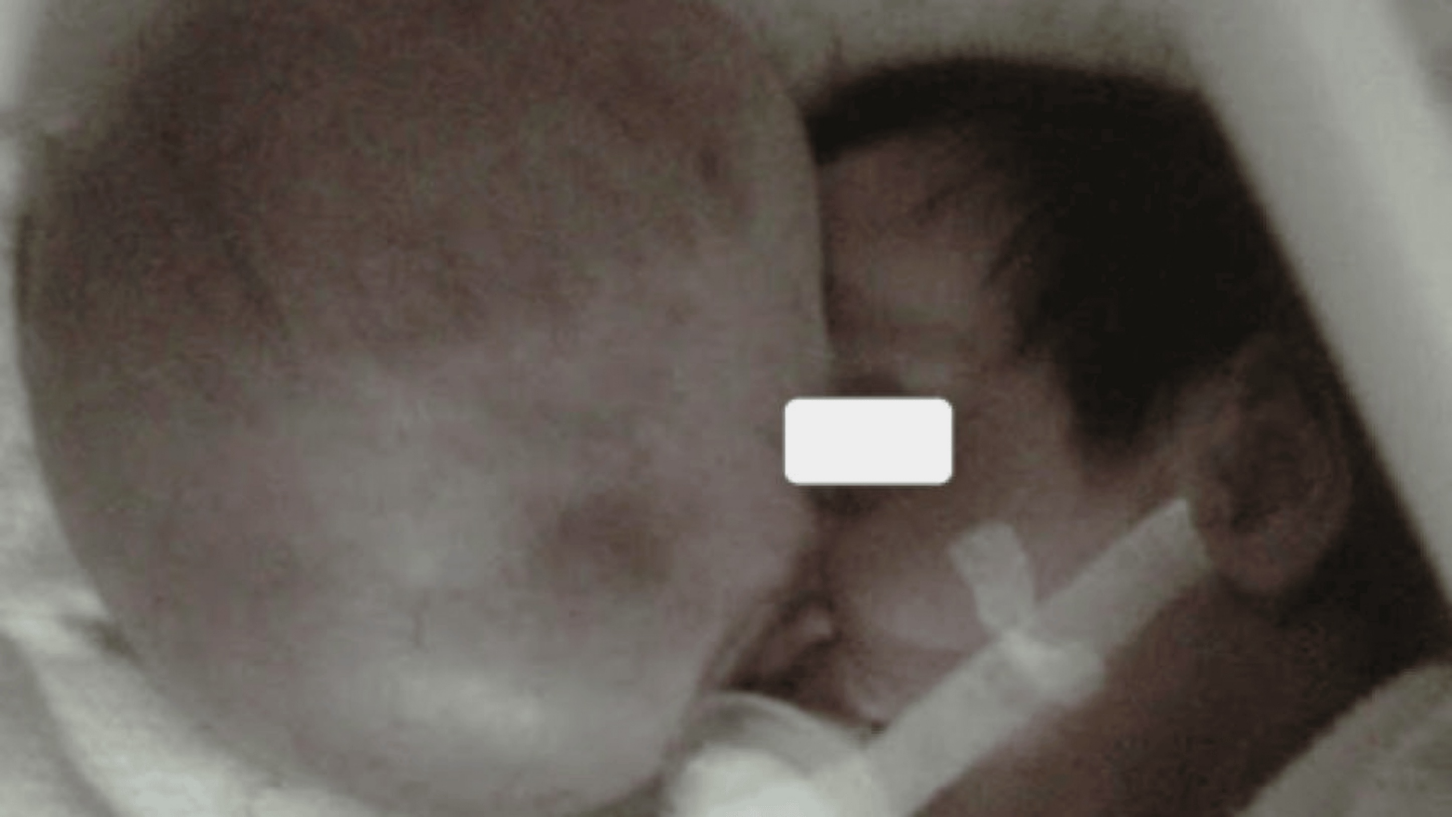
There is a tumor in the frontoethmoidal region.

A three-month-old female infant was diagnosed with giant frontoethmoidal encephalocele (Figure 1) with poor prenatal control and with no infections or risk factors reported during gestation. At the time of medical attention, the patient's developmental, psychomotor, and staturoponderal status was normal. Motorically, she could voluntarily bring her hands to objects to grasp them, socially, she was interested in people and objects in the environment, and sensorially, she moved her head to the side from where she was called. 

**Figure 2:**
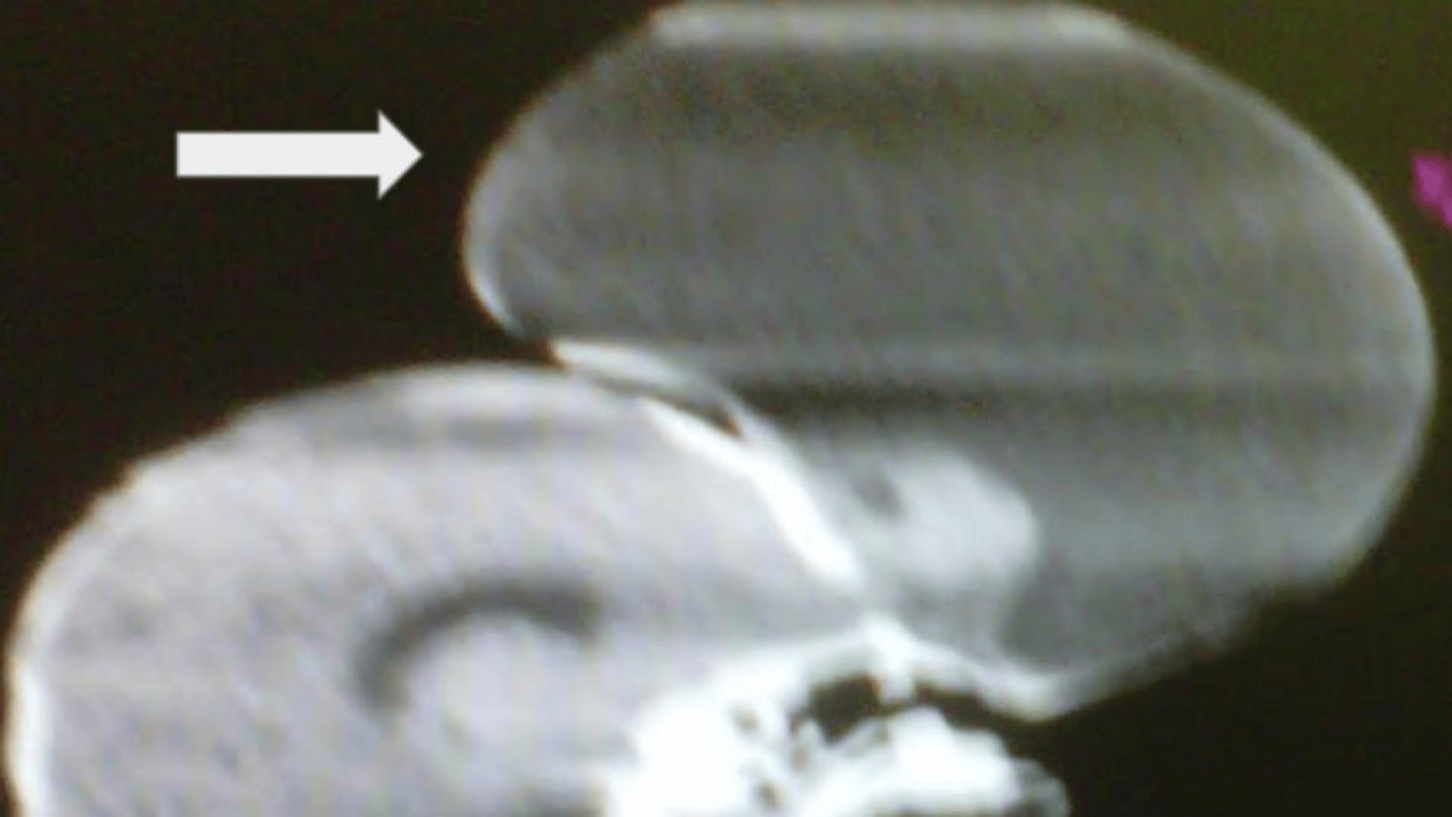
Cranial tomography shows the presence of a frontal tumor of approximately 15 cm in its major axis. Presence of communication with the meninges and soft tissue is observed.

A cranial tomography was performed, which showed the presence of a frontal tumor of approximately 15 cm in its major axis, with the presence of communication with the meninges and soft tissue (Figure 2). 

**Figure 3:**
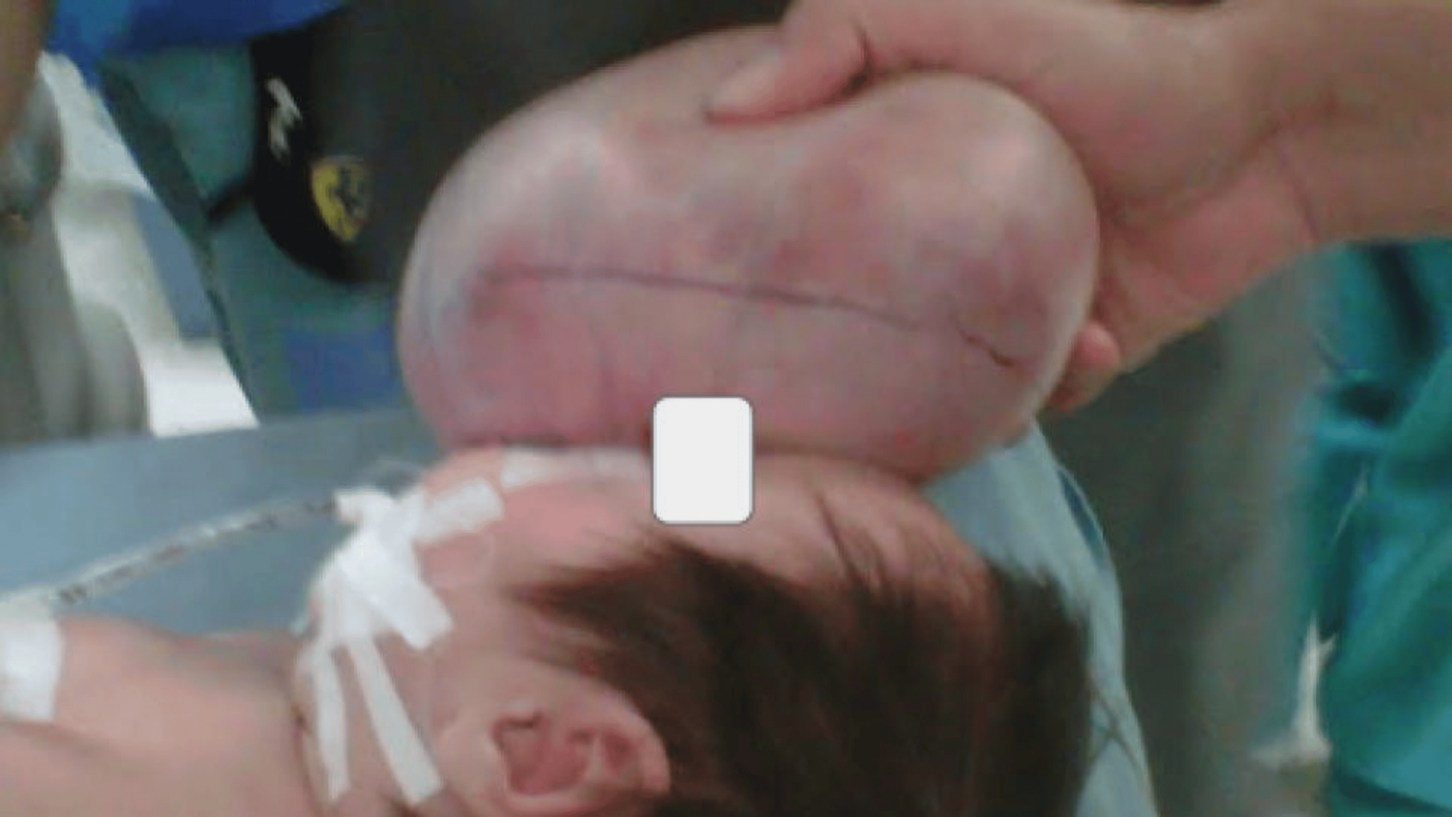
The tumor occupies the entire frontoethmoidal region.

**Figure 4:**
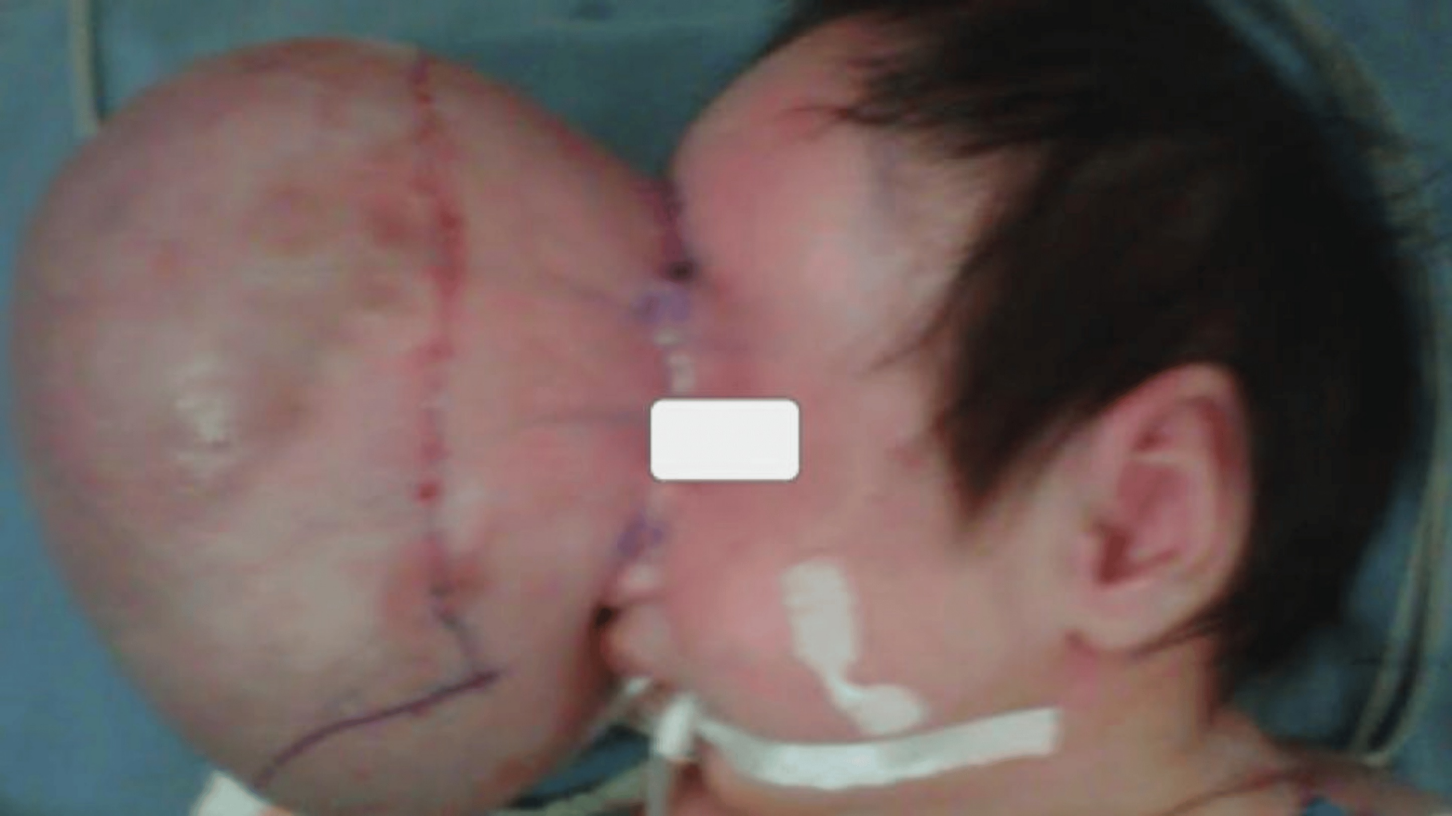
Surgical marking of the tumor is performed prior to the incision by the plastic surgery team.

Surgery was planned by a neurosurgeon and a plastic surgeon to correct the craniofacial deformities of the hard and soft tissues. The neurosurgery team performed the first approach, removing the tumor with the closure of the meninges and skull, and then the plastic surgery team, in the second surgical time, performed the craniofacial reconstruction. Using the same skin without the need to make flaps of any other structure, a primary closure was performed with adequate distribution of the tension of the skin and subcutaneous cellular tissue. The reconstruction was performed in two planes, with resorbable suture for the subcutaneous cellular tissue and simple stitches with non-absorbable suture for the skin. In total, there were approximately four hours of surgery, without complications (Figures 3-4).

**Figure 5:**
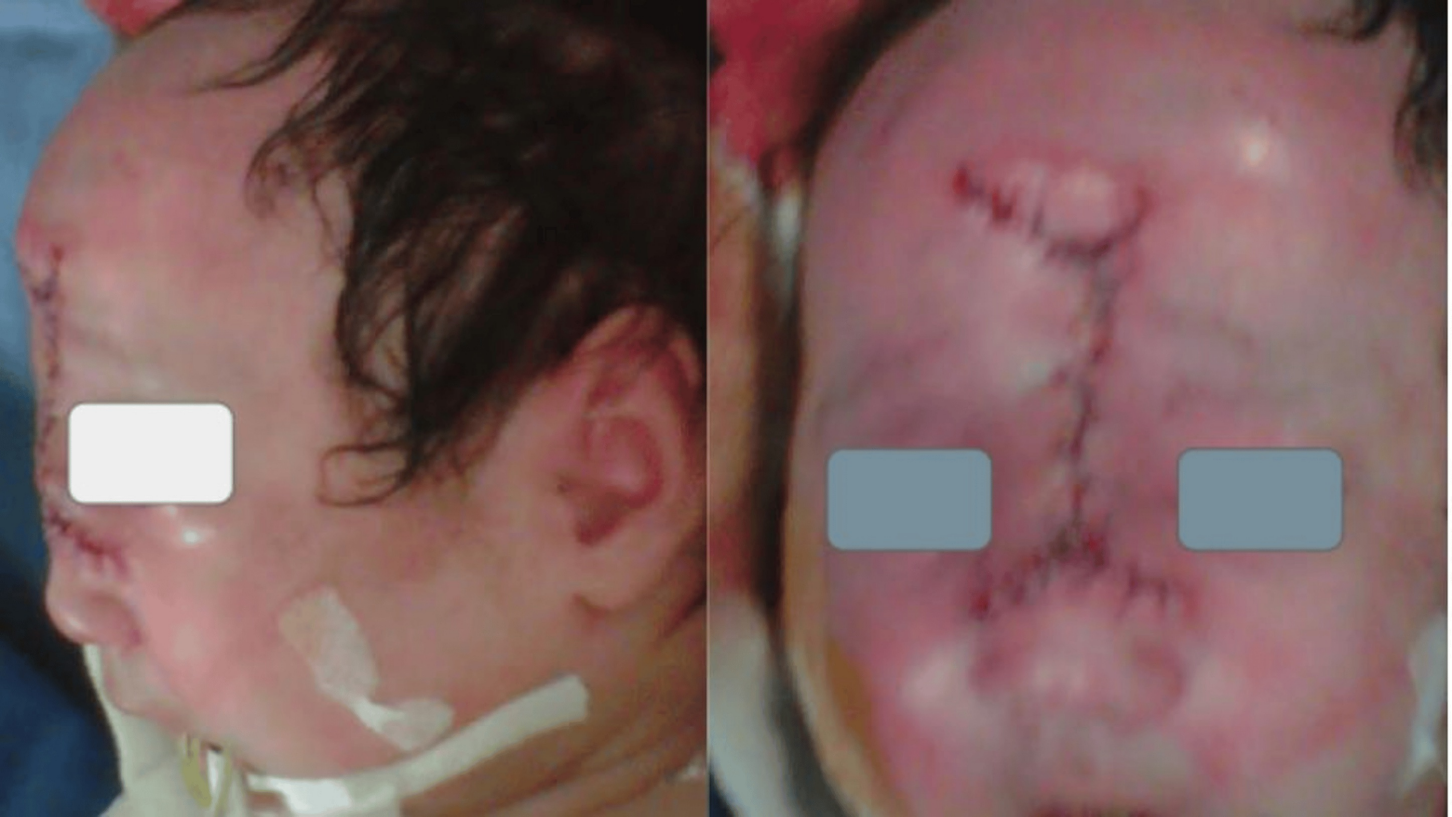
During the immediate postoperative period, the surgical wound is well managed.

**Figure 6:**
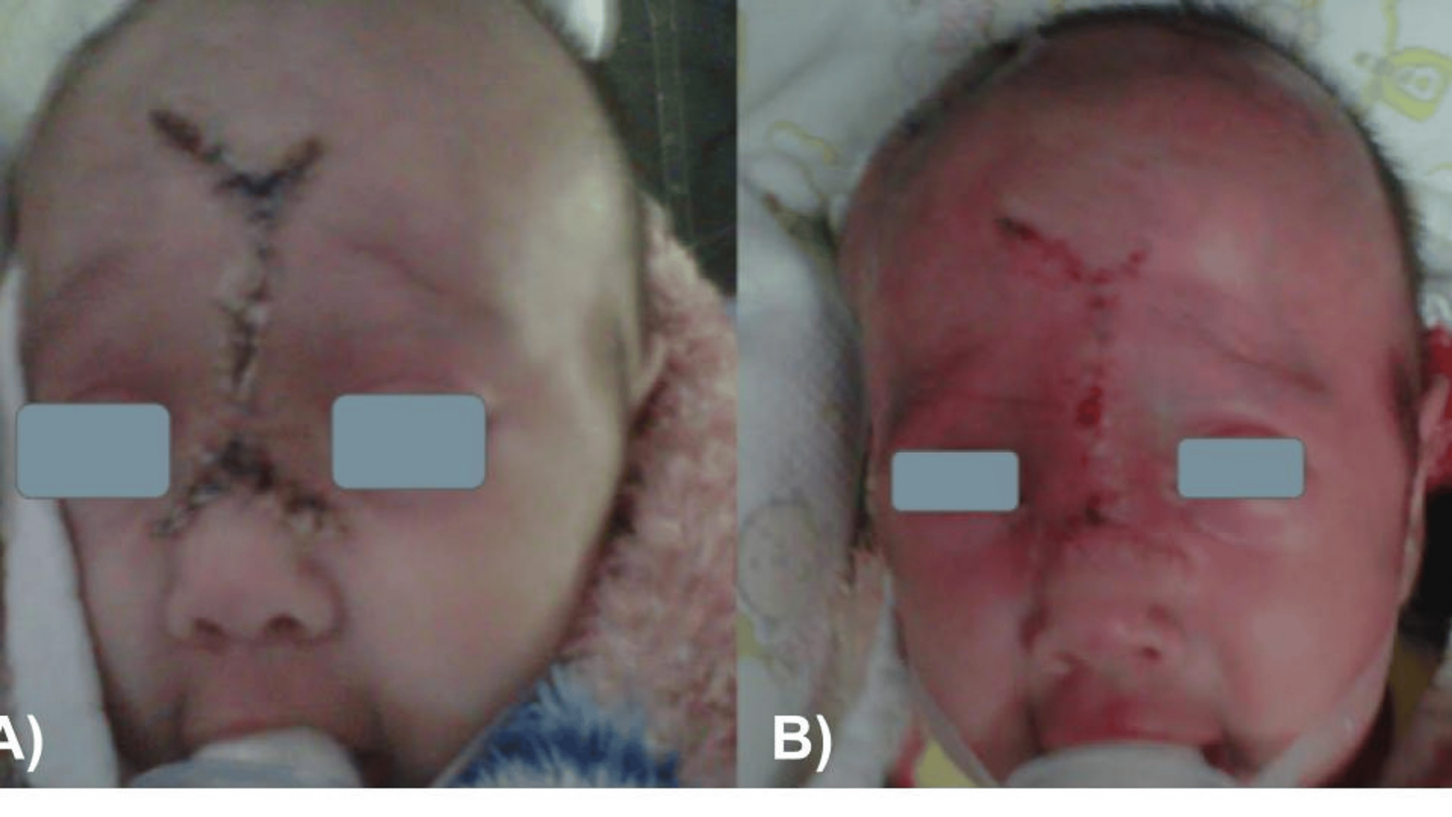
Evaluation of the patient at 15 (A) and 17 (B) postoperative days, without complications.

During the immediate postoperative period, the patient remained stable with vital signs within normal parameters and was transferred to the neonatal care unit without aminergic support and mechanical ventilation (Figure 5). She recovered uneventfully and was discharged home to the care of her parents four days after surgery. In the follow-up consultation, she was evaluated at 15 and 17 postoperative days, without complications (Figure 6). 

**Figure 7:**
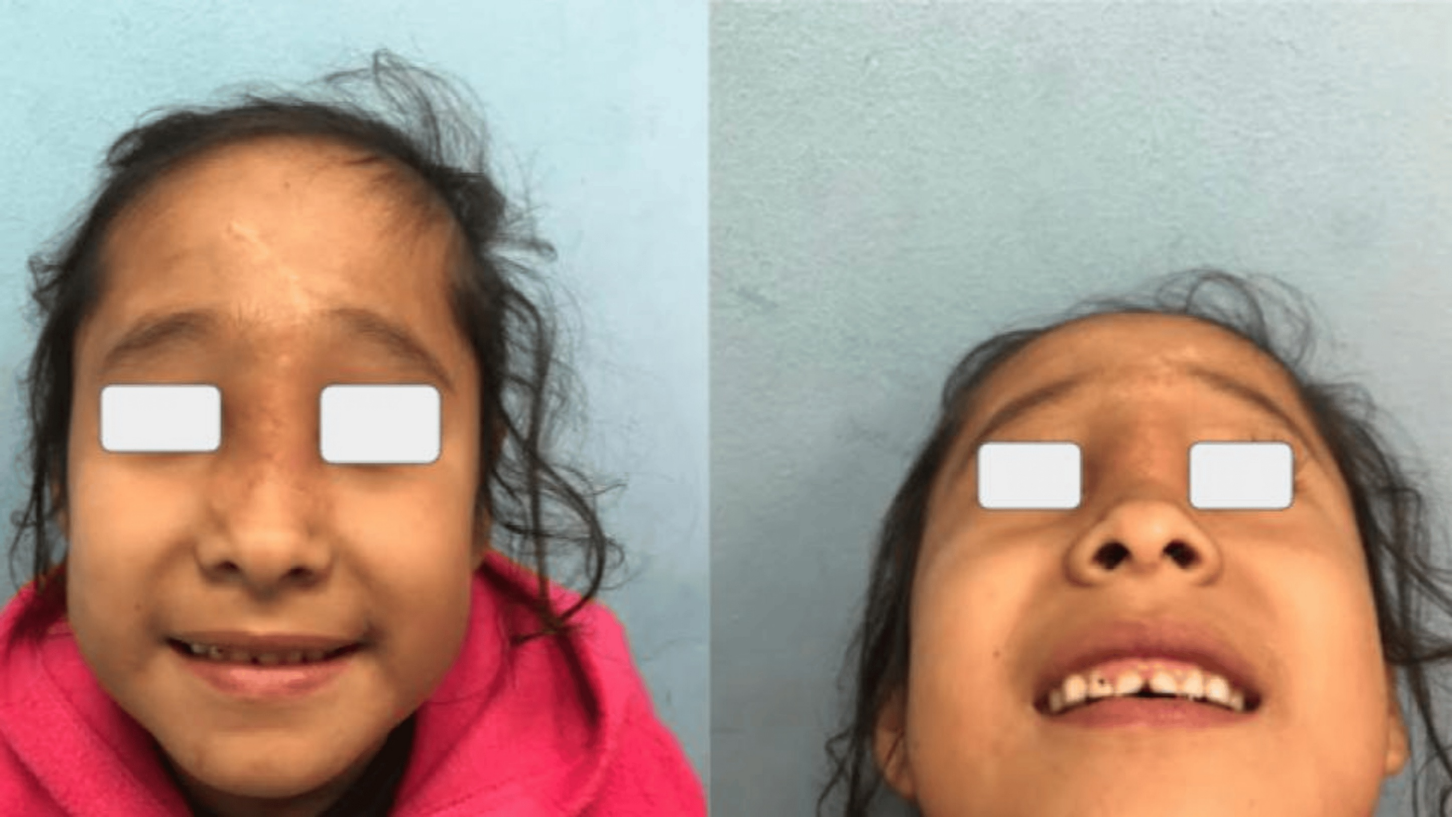
Follow-up consultation, after seven years, shows adequate aesthetic and functional results.

Currently, the patient is seven years old (Figure 7) and has an adequate quality of life for her age, without any comorbidity. She is 123 cm tall and weighs 22.8 kg which is normal for her age. She does not present an intellectual deficit, performs her physical activities adequately, does not consume any medication, and has adequate aesthetic results.

The patient's mother consented to have the child's identity revealed, and a written and signed consent statement was provided to the journal.

## Discussion

Giant encephalocele is a rare pediatric surgical entity that presents unique challenges. Few cases are reported in the medical literature, and its cause is unknown; however, clinical evidence strongly suggests that it is an autosomal recessive inherited disorder. There are factors that increase the likelihood of developing this pathology, namely, radiation which is associated with microcephaly; infections such as toxoplasmosis, rubella, or cytomegalovirus; hyperinsulinemia which is associated with craniofacial malformations; vitamin deficiencies which are associated with neural tube closure defects; and maternal smoking, alcohol, and anticonvulsants which are associated with craniosynostosis and craniofacial alterations [9-11]. 

The relationship observed in the literature is that frontal encephaloceles are more frequent in males, while 70% of posterior encephaloceles occur in females. There may be other associated anomalies, such as hydrocephalus, Dandy-Walker cyst, Chiari malformation, craniosynostosis, and microcephaly. Frontoethmoidal encephaloceles have three variants, nasofrontal, nasoethmoidal, and nasoorbital, with the nasoethmoidal encephalocele being the most frequent, while the nasoorbital encephalocele is the least frequent [12,13]. Magnetic resonance imaging (MRI) and computed tomography (CT) are necessary to plan surgery, MR angiography is also useful to assess the position of the anterior cerebral arteries, as they sometimes herniate into the sac, CT with 3D reconstruction helps to accurately define the bony defect, and angiography is only recommended when significant vessels are suspected within the encephalocele [13,14]. 

In 1976, Tessier brilliantly described a classification based on anatomy, where he assigned a correlative number to each malformation based on its location in relation to the sagittal midline. This classification was accepted internationally allowing concise and effective communication. For better surgical guidance, the orbit is divided into two hemispheres, with everything below the lower eyelid corresponding to facial fissures and what is above the upper eyelid to cranial fissures. This case can be classified as a facial and cranial cleft alteration, since the alteration of this pathology is located in the frontal and nasoethmoidal region [15]. The excess tissue can be used to design a flap, orienting the final scars along the natural lines [15,16]. 

Treatment of encephalocele is always surgical. The goal is to repair the bony defect with a tight dural closure, remove the excess skin, and remove the non-functional brain tissue. Craniofacial reconstruction is necessary for extensive frontal cases to correct hypertelorism and bony defects, and the dural defect can be repaired using the pericranium as a graft [16]. The sac is separated from the flap, and care should be taken to identify the contents. Sometimes, the sagittal torcular sinus and transverse sinus are in the vicinity of the sac, so care should be taken. The time to perform surgery depends on the size, location, associated complications, and whether a layer of skin covers the encephalocele. If there is a layer of skin acting as a protective covering, surgery may be delayed for a few months or years; otherwise, surgery is recommended soon after birth [17]. 

The most common complications are cerebrospinal fluid leakage and infection. This leakage may persist for a few days and sometimes resolves with a meningeal suture. Meningitis is a serious complication and should be treated with antibiotics [15,16]. The size of the encephalocele itself is not a guide to prognosis. As most of these children die from complications such as infection or severe respiratory involvement, therefore, individualizing the indication for surgery is the best way to treat these patients [14,15,18].

## Conclusions

We present one of the few cases of a giant frontoethmoidal encephalocele reported in the literature. These cases can endanger the child's life or leave unrecoverable sequelae such as intellectual deficit. Most children suffering from this pathology will need multiple and complex operations to try to make their facial appearance as adequate as possible. The basic principles of reconstructive surgery should be applied to obtain a good functional and aesthetic result. This patient was surgically operated on at an early age to avoid significant functional sequelae and promote the normal development and growth of the child.
